# RNA or DNA? Revisiting the Chemical Nature of the Cenancestral Genome

**DOI:** 10.1007/s00239-024-10194-9

**Published:** 2024-08-15

**Authors:** Wolfgang Cottom-Salas, Arturo Becerra, Antonio Lazcano

**Affiliations:** 1 Posgrado en Ciencias Biológicas, UNAM, Cd. Universitaria, 04510 Mexico City, CDMX Mexico; 2 Facultad de Ciencias, UNAM, Cd. Universitaria, Apdo.Postal 70-407, 04510 Mexico City, DF Mexico; 3 Escuela Nacional Preparatoria, Plantel 8 Miguel E. Schulz, Universidad Nacional Autónoma de México, Mexico City, Mexico; 4 El Colegio Nacional, Donceles 104, Centro Histórico, 06020 Mexico City, CP Mexico

**Keywords:** Cenancestor, AlkB, Demethylation repair, DNA repair, LCA genome, Great oxidative Event, Substrate ambiguity, Tertiary structure-based phylogenies

## Abstract

One of the central issues in the understanding of early cellular evolution is the characterisation of the cenancestor. This includes the description of the chemical nature of its genome. The disagreements on this question comprise several proposals, including the possibility that AlkB-mediated methylation repair of alkylated RNA molecules may be interpreted as evidence of a cenancestral RNA genome. We present here an evolutionary analysis of the cupin-like protein superfamily based on tertiary structure-based phylogenies that includes the oxygen-dependent AlkB and its homologs. Our results suggest that the repair of methylated RNA molecules is the outcome of the enzyme substrate ambiguity, and doesn´t necessarily indicates that the last common ancestor was endowed with an RNA genome.

**Supplementary Information:**

The online version contains supplementary material available at 10.1007/s00239-024-10194-9.

## Introduction

Following the publication of Oparin's ideas on the heterotrophic origin of life (Oparin 1938), it was generally assumed that the evolution of cells could be described as a linear sequence of events in which microbes became increasingly complex. This somewhat simplistic narrative was challenged, first, with the proposal of the endosymbiotic origin of eukaryotes (Sagan 1967) and, secondly, when the evolutionary comparison of the sequences of the ribosomal small subunit RNA (16/18S rRNA) led to the construction of an unrooted, trifurcated phylogenetic tree, which all species can be grouped in one of three cell lineages or urkingdoms. These were originally termed eubacteria, archaebacteria and urkaryotes, which corresponded to the eukaryotic nucleocytoplasm (Woese and Fox 1977). Following a period of rather intense taxonomic debates (Mayr 1998; Woese 1998), these lineages were recognised as three major cell domains, now termed as Bacteria, Archaea and Eucarya (Woese et al. 1990).

The scientific impact of the use of rRNA as a phylogenetic marker can hardly be overestimated. It made it possible to recognise the three major cell lineages and, in particular, that prokaryotes are divided into two ancient, monophyletic groups. What is somewhat surprising is the persistence of the discussions on the nature of the last common ancestor of Bacteria, Archaea and Eucarya, for which Woese and Fox (1977) coined the term progenote. As demonstrated by the papers included in this volume, although nearly half a century has passed, the disagreements on the biological nature of the progenote and of the last common ancestor remain largely unresolved. These discussions, however, have led to fruitful debates on very early phases of cellular evolution which had been largely ignored.

### What is the Progenote?

Although the basic patterns of DNA replication and gene expression are the same for Bacteria, Archaea and Eucarya, recognition of their differences led Woese and Fox (1977) to conclude that they were the evolutionary outcome of independent evolutionary refinements, i.e. that each of these three lineages had evolved independently from the progenote, a hypothetical entity in which phenotype and genotype still had an imprecise, rudimentary linkage relationship. While they recognised that eukaryotes had descended from prokaryotes, they wrote that this relationship of descent is defined “in the sense that the prokaryotic is an organisational, not a phylogenetic distinction. In analogous fashion prokaryotes arose from simpler entities. The latter are properly called progenotes, because they are still in the process of evolving the relationship between genotypes and phenotype” (Woese and Fox 1977; see also Kandler 1994; Gogarten 2019).

Since the 16/18 S rRNA-based phylogeny is an unrooted tree, in Woese and Fox (1977) proposal the progenote represents the last common ancestor, because it was the hypothetical ancestral entity located at the trifurcation that led to the Bacteria, Archaea and Eucarya. In Woese and Fox original scheme it also corresponds to the root of the tree, but they never claimed that it was the first living organism, and nor did they address the presence or absence of membranes, its metabolic abilities, or the environment in which it originated and thrived. According to the original definition however, it was an entity in which a primitive error-prone form of genetically encoded protein biosynthesis had already evolved.

The suggestion that the progenote was endowed with an RNA genome was implicit and well accepted, since the likelihood that in early stages of evolution RNA genetic polymers had preceded extant cellular DNA genomes had already gained wide acceptance (Lazcano 2012). Indeed, Woese (1967) had explicitly acknowledged the primeval role of RNA molecules in his influential book *The genetic code: the molecular basis for gene expression*, which can be read as an evolutionary account of translation based on the idea that the highly refined structures of extant ribosomes and specificities of aminoacyl tRNA synthases could have not existed during the early stages of evolution (Lazcano 2012).

The most significant breakthrough in the search for the cenancestor was the rooting of the tree of cellular life. The first to suggest the use of duplicated genes as outgroups to root a tree was Dayhoff (1972). This methodology was successfully employed by Gogarten et al. (1989) and Iwabe et al. (1989), and corroborated by numerous analyses including those of Brown and Doolittle (1995) and Gribaldo and Cammarano (1998), all of which placed the root of the rRNA tree in the Bacteria, i.e. the bacterial phenotype is the oldest one, which also implies that the eukaryotic nucleocytoplasm evolved from the archaea.

Placing the root in the Bacteria implies that the earliest recognisable evolutionary split occurred in prokaryotes, and by pushing back in time the progenote a clear distinction between it and the last common ancestor was established. Accordingly, the reconstruction of the gene complement of the LCA can be limited to the comparison of homologous trait shared by the Bacteria and the Archaea, and does not need to include the Eucarya. However, as summarised in Table 1, the characterisation of the last common ancestor, which is located in the earliest split in universal phylogenies, is far from solved. Indeed, the plurality of terms that have been coined to describe the last common ancestor (LCA), including cenancestor, urnacestor, commonote, last universal common ancestor, last universal cellular (LUCA,) and others, reflects the different and sometimes opposing views on its nature (Becerra et al. 2007). Here we adopt the term cenancestor proposed by Fitch and Upper (1987). The purpose of this paper is to address the issue of the chemical nature of the LUCA genome, i.e. whether the population ancestral to both Bacteria and Archaea was already endowed or not with a double-stranded DNA genome.

**Table 1:** Summary of proposals on the chemical nature of the cenancestor genome. Papers that do not explicit address this issue have not been included. See text for their critical analysis

Proposed nomenclatures	Methodologies	Chemical nature of the cenancestral genomes	References
Progenote	Qualitative assessment based on the differences between gene expression and replication in the three major cellular domains	Implicit assumption of an RNA genome with an imprecise linkage between genotype and phenotype	Woese and Fox (1977) Woese (1987)
Last common ancestor (LCA)	Qualitative assessment of traits shared between the three cellular domains	Extant prokaryotic-like DNA genome	Lazcano et al. (1992)
Last common ancestor (LCA)	Comparison of the two strictly intracellular parasites *H.influenzae* and *M. genitalium* genomes	RNA genome encoding 256 genes	Mushegian and Koonin (1996)
Last common ancestor (LCA)	Protein sequence comparisons	RNA/DNA genome with DNA produced by reverse transcription	Leipe et al. (1999)
Last universal common ancestor (LUCA)	Qualitative assessment of LUCA’s traits through a variety of comparative methodologies	Mixed RNA/DNA replication system with 500–600 genes	Koonin (2003)
Last common ancestor (LCA)	Comparative analysis of conserved genes in COGs database	Extant prokaryotic-like DNA genome	Harris et al. (2003)
Last universal common ancestor (LUCA)	Qualitative assessment of putative RNA repair mechanisms	Possible RNA-like genome	Poole and Logan (2005)
Last common ancestor (LCA)	Analysis of conserved proteins in extant free living prokaryotes	Double-stranded DNA genome	Delaye et al. (2005)
Last universal common ancestor (LUCA)	Qualitative assessment of hypothetical LUCA’s physiology in a hydrothermal environment	Viral-like fragmented RNA genome	Koonin and Martin (2005)
Last universal common ancestor (LUCA)	Qualitative assessment of LUCA’s physiology	DNA as a stable repository with RNA-based replication	Weiss et al. (2018)

### The Cenancestral Genome

To the best of our understanding, the first attempt to characterise LUCA was developed by Lazcano et al. (1992). The characteristics of an ancestral organism can be inferred from the distribution of homologous traits in its descendants, but 30 years ago only few genes present in the three cellular lineages had been sequenced and no completely sequenced microbial genomes were available. As a result of an extensive and painstakingly review work (Lazcano et al. 1992), it was possible to compile a catalog of traits common to the three cell lineages that indicated that the ancestral phenotype already possessed (a) a translational apparatus with an oligomeric RNA polymerase, complex ribosomes endowed with ribosomal proteins; (b) a membrane-associated H + ATPase engaged in active transport; and (c) a complex set of metabolic enzymes, including the histidine biosynthetic pathway. This inventory suggested that the last common ancestor was not a protocell or any other pre-life progenitor system, but a population of organisms very much like extant prokaryotes (Lazcano et al. 1992; Becerra et al. 2007). It also implies that the basic genetic processes and major subcellular molecular traits have remained largely unchanged since the early Precambrian, perhaps due to the intricate character of the networks of gene interactions and the complex relationships between deficient metabolic processes (Lazcano and Miller 1996).

The study of sets of paralogous sequences like the hydrophilic subunits of ATPases, elongation factors, and a rather large number of metabolic enzymes can also provide specific insights into the biological events that took place prior to the appearance of the cenancestor (Lazcano et al. 1992). As summarised elsewhere, the high numbers of these duplicates implies that the LCA descended from simpler cells in which only one copy of each of these genes existed (Lazcano 1995). The numerous paralogous gene families support the hypothesis that primitive enzymes were unspecific catalysts that could react with a wide range of chemically related substrates, which can be seen as a mechanism by which primitive cells with small genomes could overcome their limited coding abilities (cf. Becerra et al. 2007).

The similarities shared by the ancient proteins present in all three lines of descent also implies that considerable fidelity already existed in the last common ancestor genetic systems (Lazcano et al. 1992). No such preservation of sequence information is found in RNA genomes (Reanney 1982; Hernández-Morales et al. 2022), which suggests that double-stranded DNA genomes were a trait that had evolved prior to the divergence of the Bacteria and Archaea (Lazcano et al. 1992; Becerra et al. 2007). However, reconstruction of ancestral states is a treacherous terrain riddled by the vagaries of convergence, loss of sequences, lateral gene transfer, sample size and other methodological issues (Becerra et al. 1997, 2007; Estrada et al. 2022). It is therefore not surprising that not all accept the conclusion that the LCA population was endowed with DNA genome.

Confusion still abounds in the field, as shown by the relatively recent suggestion in which the LCA is equated with the first living entities (Weiss et al. 2016). Proposals that the LCA had a RNA or a hybrid RNA–DNA genome have been based on indirect evidence that include (a) the absence of ribonucleotide reductases in the theoretical reconstruction of minimal cells based on the comparison of two parasitic bacteria with highly streamlined genomes (Mushegian and Koonin 1996); (b) the apparent unrelatedness of the bacterial DNA polymerases and primases with their archaeal/eucaryal counterparts (Leipe et al 1999); (c) the absence of homologous DNA-replication machinery in LCA gene-sets (Koonin 2003); and (d) the eucaryal fragmented genome, as indicated by separate chromosomes, and intense intranuclear processing of RNA transcripts (Penny and Poole 1999), together with reports on enzyme-mediated repair of RNA adducts (Poole and Logan 2005). Prompted by reports on the in vivo repair of methylated adenine and cytosine in RNA molecules by AlkB oxidative demethylases, Poole and Logan (2005) have raised the possibility that LUCA was endowed with an RNA genome. The purpose of this paper is to critically analyse this hypothesis, and to argue that in some cases biogeochemical considerations may assist in the identification and proper interpretation of cenancestral traits. We also present three-dimensional structure-based phylogenies as a complementary and more robust approach to gain insights into the early evolution of O_2_-dependent DNA repair mechanisms, and their application for a better understanding of the chemical nature of the LCA/LUCA or cenancestral genome.

### Oxygen and Nucleic Acid AlkB-Mediated Demethylation Repair

Following the emergence of life, the second most important biogeochemical event on Earth was the origin and accumulation of a permanent oxygen-rich atmosphere, a process that took place well after LUCA, i.e. long after the evolutionary split of Bacteria and Archaea ~ 3460 Gyr ago (Ueno et al. 2006; Becerra et al. 2007). Several major evolutionary consequences of the availability of free oxygen can be recognised, including (a) the development of defence and repair mechanisms such as MsrB (Delaye et al. 2007) and AlkB (Falnes et al. 2002) repair mechanisms; (b) the emergence of O_2_-dependent enzymes and routes that took advantage of the oxygen potential for new reactions; and (c) the evolutionary development of O_2_-sensing mechanisms like hypoxia induced factors (HIFs) and oxygen transport molecules like hemerythrins (Raymond and Segré 2006; Khademian and Imlay 2021; Alvarez-Carreno et al 2016; Khersonsky and Tawfik 2010).

One of the most successful folds that took advantage of biochemical possibilities opened by the availability of free oxygen is the cupin fold (also known as the double-stranded-beta-helix fold DSBH). The cupin fold caught the attention of researchers 40 years ago, when the key role of the AlkB protein in the repair of alkylated DNA (Falnes et al 2002) was identified in *Escherichia coli* (Kataoka et al. 1983). Based on iterative PSI-BLAST searches, Aravind and Koonin (2001) identified AlkB as a 2-oxoglutarate (2OG) and Fe (II)-dependent oxygenase.

The 2OG-Fe (II) oxygenases (hereinafter 2OGxs) are part of one of the most diverse pfam extant clans. As of today, the CL0029 “cupin” superfamily includes 72 protein families with thousands of different domain architectures. This remarkable diversity of sequences and architectures is reflected in a considerable number of functions (Ougland et al. 2015), and a low level of sequence identity/similarity among the 72 families (Agarwal et al. 2009).

This diversity represents a challenge for the study of the molecular evolution and phylogenetic distribution of the cupin fold. Attempts to overcome the limits of traditional alignments and phylogenies have led to other methodologies like sequence clustering (Mielecki et al. 2012), secondary structure prediction analysis and structure-based phylogenies. The outcome of 3D-structure analysis for AlkB homologues was reviewed by Dunwell et al. (2001), while Agarwal et al. 2009 performed a structure-bases phylogeny with proteins containing a cupin fold suggesting that, given the high divergence among the fold, a 3D-based analysis may help to assign new unknown functions for new homologs.

### The Success and Complexity of the Cupin Fold

**Fig. 1:**
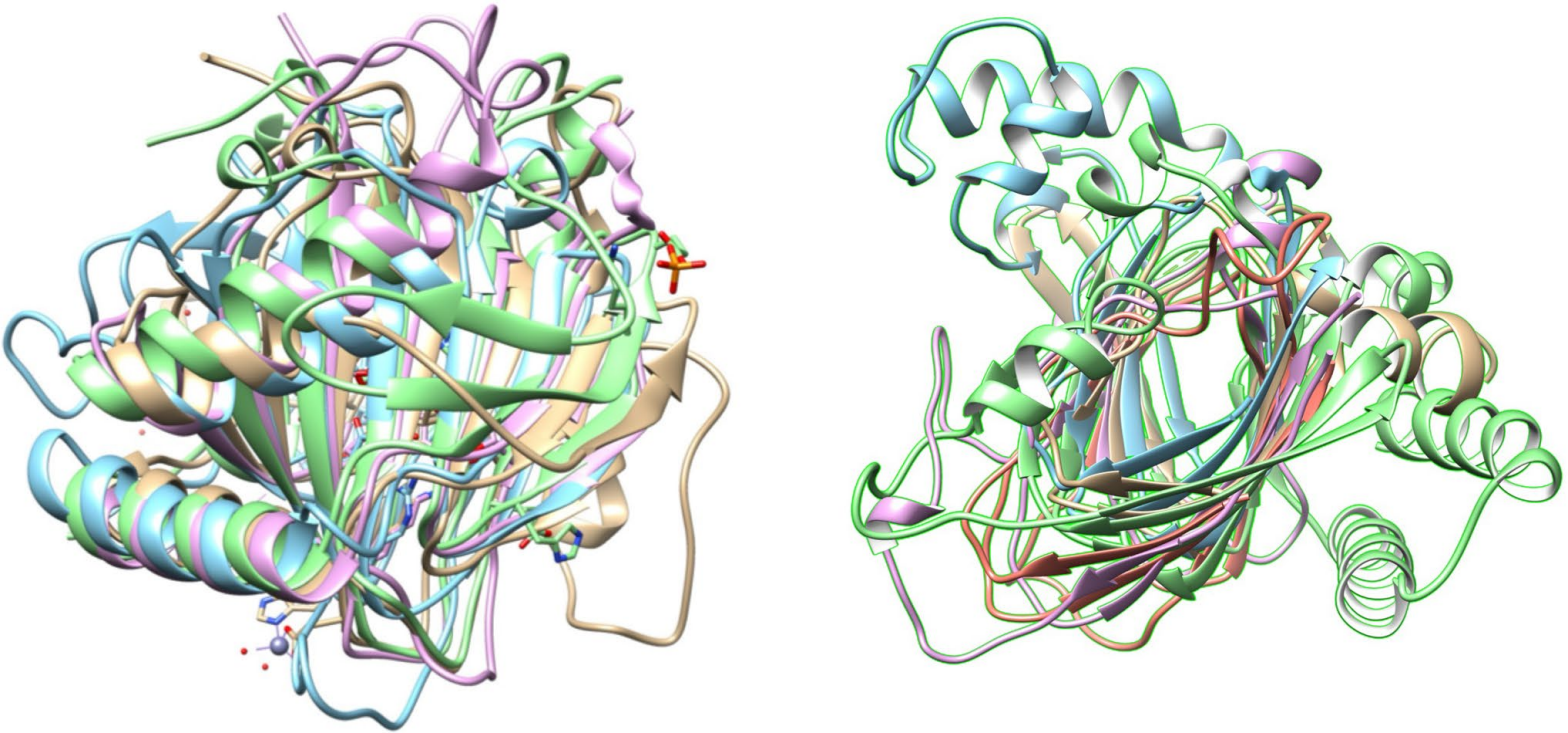
Structural comparison of five CL0029 pfam clan members representing the cupin fold as a typical DSBH topology. PBD codes: 1bk0_2OG-FeII_Oxy (pink), 5j3u_cNMP_binding (gold), 5nla_AraC_binding_2 (green), 6ip0_JmjC (cyan) and 6t8m_2OG-FeII_Oxy_3 (orange). The image was processed with UCSF-Chimera software using matchmaker algorithm with 5j3u_cNMP_binding structure as reference (Color figure online)

The cupin fold (also known as jumonji C (JmjC) or jelly-roll fold) is part of a protein superfamily that exhibits an extraordinary diversity of functions and a characteristic core with a double-stranded-beta-helix (DSBH) topology, that can exhibit some structural variations (Khuri et al. 2001; Islam et al. 2018) (Fig. 1). As of today 72 protein families have been described as members of the superfamily cupin, that includes a wide number of functions including isomerases or epimerases acting in cell wall carbohydrates, storage proteins in plants, transcription factors in animals (Dunwell et al. 2001), posttranslational modifications involved in collagen biosynthesis, lipid metabolism, nucleic acid repair, and chromatin and RNA splicing (Aik et al. 2012). It is well known that the variations in intermotifs in cupin folds (i.e. insertions in loops) make the identification of conserved residues a challenge (see, for instance, Fig. 1) (Khuri et al. 2001).

Since the fold comprises non-enzymatic as well as enzymatic proteins (Dunwell et al 2001), that catalyse between 50 and 100 different biochemical reactions, making this fold one of the largest and most catalytically diverse superfamily (Dunwell et al 2004), with approximately 5000 different architectures endowed with at least one cupin fold member, and over two million different proteins that have a cupin fold homolog (Paysan-Lafosse et al. 2023). In particular, the cupin proteins that act like DNA repair enzymes are all 2OGxs-like present in bacteria and some viruses (Hausinger 2015). In general, the reaction catalysed by 2OGxs involved in DNA repair depend on free molecular oxygen for oxidative catalytic reactions of S_N_2 alkylating agents in a direct reversal reaction (Begley and Samson 2003).

### The cNMP_Binding Domain as the Ancestral Cupin-Like Fold

Ribonucleotide- and modified ribonucleotide-binding domains are an ancient feature whose origin may be traced to a stage in which RNA and ribonucleotides and modified ribonucleotide played a more conspicuous role in basic biological processes (Hernandez-Morales et al. 2019). Many folds that bind modified ribonucleotides (mono, di or cyclic-ribonucleotides), like the cNMP_binding domain, have a $\beta$-sheet fold that exhibits different interactions and binding patterns. Analysis of the evolution of lactate dehydrogenase, an enzyme with AMP/NMN binding units, led to the proposal that a $\beta$-sheet with three parallel strands corresponds in act to a primordial mononucleotide binding fold (Rossman et al. 1974; Rossmann and Argos 1978). Indeed, the cNMP_binding domain appears to be a reasonable candidate for an ancestral cupin ancient-like fold that reflects a conserved ancestral binding function that may have originated in a RNA/protein world.

### The Biogeochemistry of O_2_-Dependent DNA-Repair Enzymes

It is well known than many enzymes require ions as co-factors to function. Accordingly, the relative abundances and the bioavailability of these ions can provide insights of the evolutionary history of specific enzymes, which is the case of AlkB and all the 2OGxs. The relative abundance of Fe^2+^ on Earth has varied across time and has a complex history coupled with that of free dioxygen. For instance, Fe^2+^ was abundant prior to the Great Oxidation Event ~ 2.4 Gyr ago, but the reaction of dissolved oxygen in oceans oxidised Fe^2+^ and the valence state changed to Fe^3+^, causing the precipitation of insoluble Fe^3+^ oxides. This is demonstrated by Moffett (2021) analysis, where the presence of Fe^2+^ is more abundant in oxygen deficient zones where O_2_ concentrations can actually reach zero.

However, the scarcity of Fe^2+^ did not cause an “iron crisis” (Khademian and Imlay 2021), perhaps because of the low concentration requirements of this element inside cells (Semsey et al. 2006). As discussed below, the complex geochemical history of transition metals specifically of iron, restricts the possibility of some enzymatic reactions which are relevant to theoretical reconstructions of the cenancestral gene complement.

The evolution of the cupin fold is a complex issue and remains an open question. Although there is no consensus of the evolutionary history of this fold, its well-known success is reflected on its wide distribution in the three domains of life and in some viral groups. As part of an effort to date the emergence of oxygen-dependent protein families, Jabłońska and Tawfik (2021) identified a list of O_2_-dependent enzymes that includes a DNA demethylase with the 2OG_FeII_Oxy_2 cupin domain that emerged at the hypothetical Last Universal Oxygen Ancestor (LUOA) node, i.e. long after cenancestral epochs 3.8–4.2 Gya ago (Moody et al. 2024).

## Methodology

### Tertiary Structure-Based Phylogeny

**Fig. 2:**
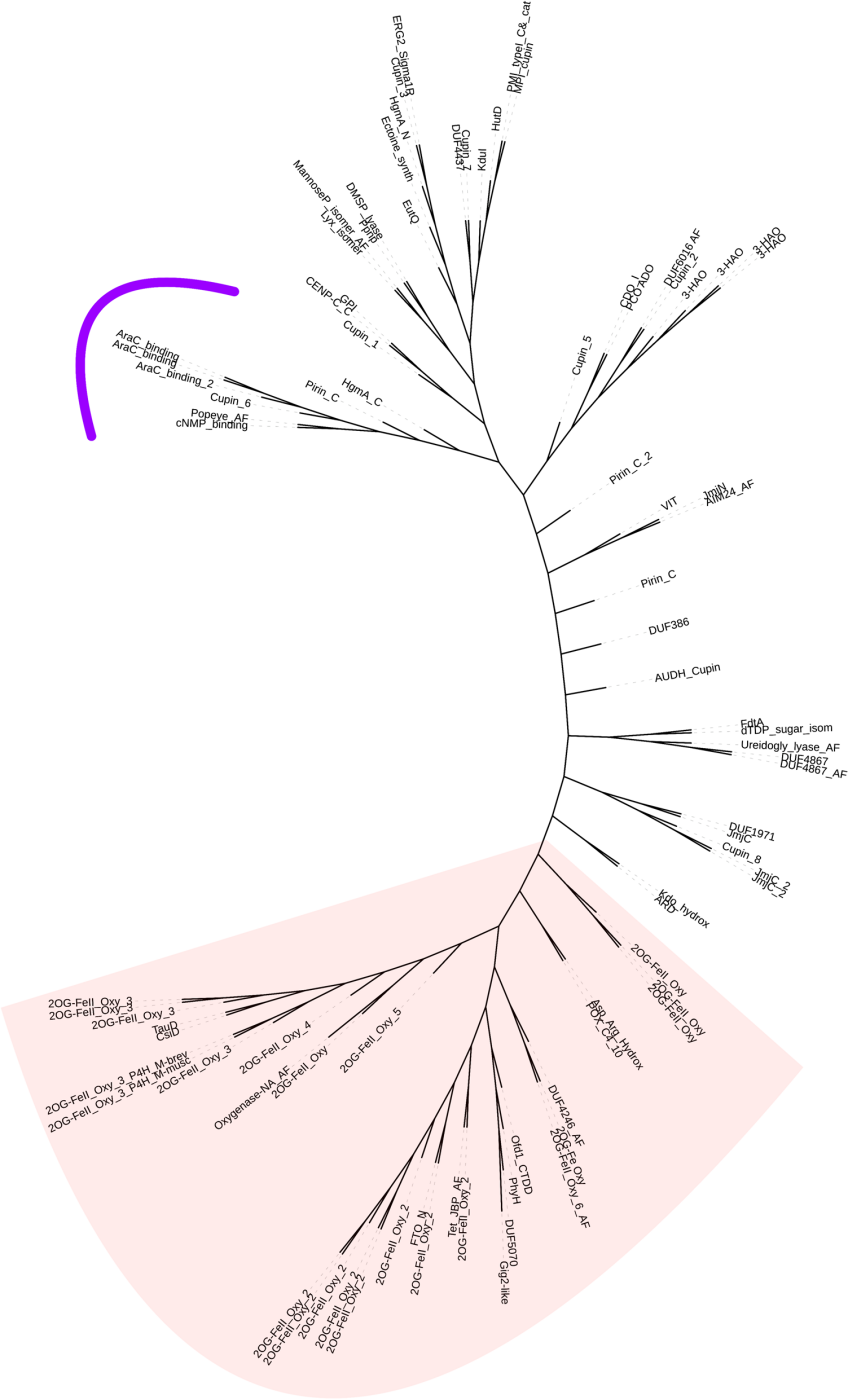
Tertiary-structure based unrooted phylogeny for the 72 members of the pfam clan CL0029. Tips surrounded by the purple line lack oxidoreductase activity and do not require free oxygen and Fe^2+^ while the non-surrounded tips exhibit a strict requirement of O_2_ and Fe^2+^. The red shadowed area indicates the monophyletic character of 2OG dioxygenases (Color figure online)

**Fig. 3:**
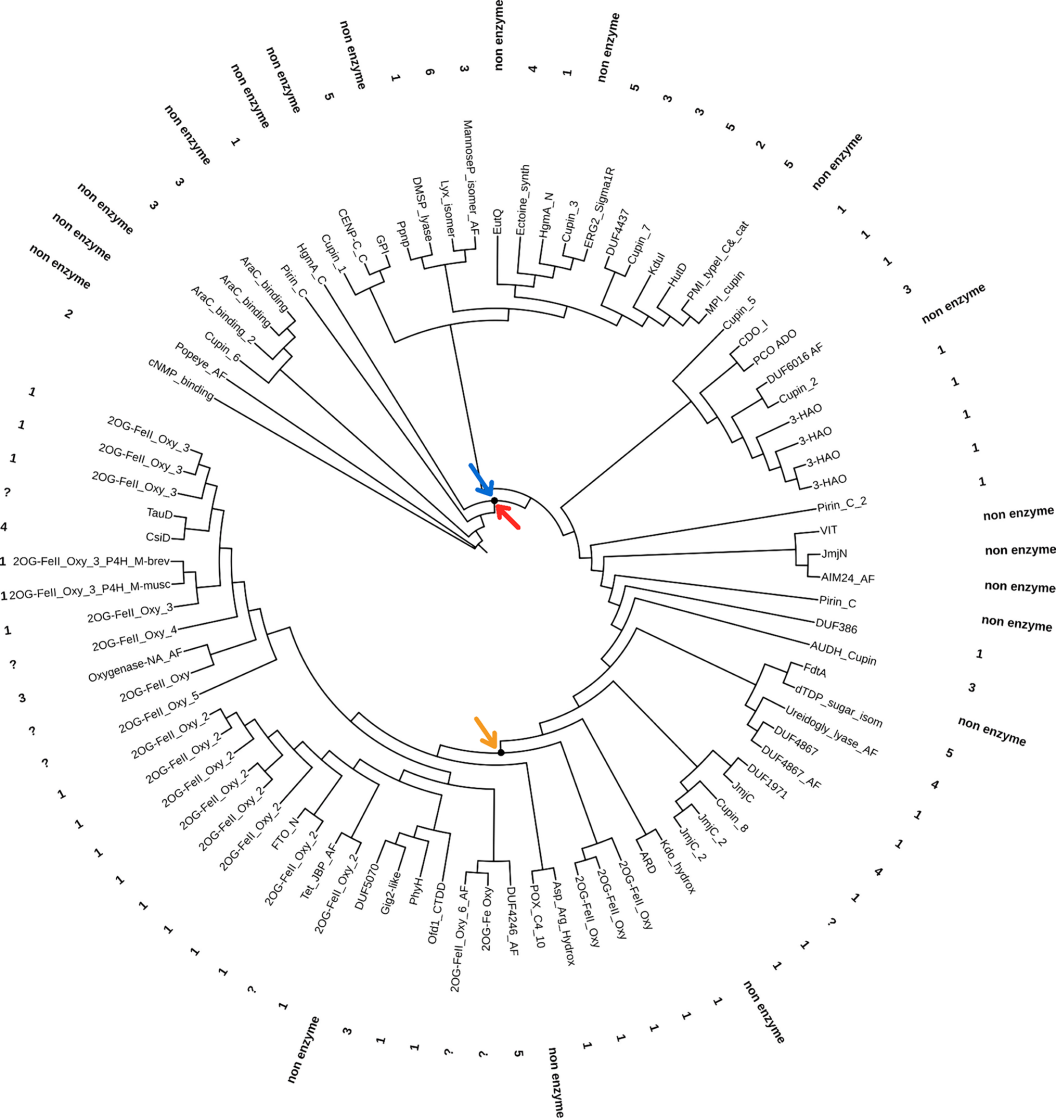
Tertiary-based rooted phylogeny of all 72 domains/families endowed with the cupin fold. The red and blue arrows represent the incorporation of O_2_ and Fe^2+^ during the evolutionary history of the fold, and the origin of the fold’s oxidative activity. The orange arrow marks the later origin of the 2OG dioxygenases. Numbers at tips represent the EC class suggested by ECpred. Terminal tips annotated as “non enzyme” indicates that non-enzymatic activity was assigned by ECpred, suggesting a structural or binding function for the protein, while a “?” at tip indicates that ECpred was unable to assign an EC class number or a non-enzymatic function (Color figure online)

From the Protein Data Bank (December 2023/RCSB-PDB) a total of 76 structures containing CL0029 members were downloaded and trimmed to obtain only the homologous cupin domains regions with no mutations and a ligand present in the structure. In addition to the resolved crystal structures analysed, the sample was completed by adding top ranked AlphaFold database predictions (Jumper et al. 2021) for nine non-resolved or mutated structures of CL0029 *pfam* clan. The trimmed structures, including the predicted and complete resolved structures were aligned, and the TM-score was calculated by pairing comparison using mTM-align software (Dong et al. 2018) The output matrix was used by Fitch-Margoliash algorithm with global rearrangement optimization in the PHYLIP package (Felsenstein 2005). Two tertiary structure-based trees were constructed, one of them unrooted (Fig. 2) and a second one rooted using the cNMP_binding domain structure as an outgroup (Fig. 3). The tree was visualised and annotated in iTOL v6 (Letunic and Bork 2024). For each tree tip, a representative sequence at Interpro-pfam database were selected and their EC number was predicted with ECpred (Dalkiran et al. 2018).

### Structure Prediction and Validation of Two Procollagen Enzymes

Because no fully resolved procollagen-proline 4-dioxygenase from metazoa is available, *Monosiga brevicollis* (A9UV67_MONBE) and *Mus musculus* (P4HTM_MOUSE) procollagen-proline 4-dioxygenases tertiary structures were predicted using RoseTTAFold (Baek et al. 2021) and validated following the methodology developed by Martínez-Castilla and Rodríguez-Sotres (2010). The CL0029 members with non-resolved structure deposited in the PDB were retrieved from AlphaFold Protein Structure Database (Jumper et al. 2021).

## Results

### Tertiary Structure-Based Phylogeny and Structural Analysis

All the structural predictions of unsolved structures of cupin domains were included in the trees shown in Figs. 2 and 3. The significance of 2OGxs domain predictions is shown, for example, in the position 2OG-FeII_Oxy_3 domain in both trees. Because this domain is a quite important part of the architecture in the procollagen-proline 4-dioxygenase, the close relation of this domain prediction to other 2OGxs makes the prediction biologically valid. Figure 2 shows an unrooted tertiary structure-based phylogeny of all the 72 members of the cupin superfamily including the subunit $\beta$ procollagen-proline 4-dioxygenase (2OG-FeII_Oxy_3 domain) for *M. brevicollis* and *M. musculus*.

Two main groups can be recognised in the unrooted phylogeny shown in Fig. 2. One of them is formed by domains that do not require neither oxygen nor iron, as the ECpred prediction suggest. Examples of these domains include the cNMP_binding domain, AraC_binding and Cupin_6 where transferase activity and a non-enzyme EC was assigned.

In the well-defined second group (Figs. 2 and 3), according to the EC number prediction, the majority of the cupin domains require molecular oxygen and iron for their enzymatic reactions. The 2OGxs forms a clearly defined monophyletic group (red shadowed area in Fig. 2), and appears to be closely related with other domains that partake in a huge diversity of reactions in which oxidoreductase is the prevalent enzymatic activity. In spite of the problems associated with the selection of an external group to root the phylogeny, Fig. 3 shows the same data but with a root in the cNMP_binding domain in an attempt to picture the evolutionary history of cupin fold evolution. Our hypothesis suggests that the first cupin domains had no oxidoreductase activity and, a cupin fold may have structural and transferases activity. The blue-red node shows where the oxygen and iron dependency first evolved and led to oxidoreductase activity, with the exception of EC 7 enzymes. The rooted phylogeny also shows consistently that all the EC numbers are part of the cupin superfamily.

## Discussion and conclusions

### The Evolutionary History of O_2_-Dependent Enzyme-Mediated Nucleic Acid Repair and the Chemical Nature of Cenancestor Genome

Several attempts based on different approaches to characterise the cenancestral genomic traits (Table 1) have concluded that there is no common DNA replication machinery. However, as shown here, important insights in the chemical nature of cenancestor genome can be based on additional information provided by repair enzymes to address this issue. An example of this approach relies on the use of the observed activity of the enzyme AlkB and its homologues.

There is a rather large amount of literature addressing the evolution of families with the cupin fold (Agarwal et al. 2009; Dunwell et al. 2001, 2004; Khuri et al. 2001). Given the complexity of the cupin superfamily evolution, it is difficult to establish the antiquity of the oxidative demethylation (Poole and Logan 2005). Fortunately, one of the most well-studied cupin superfamily members are precisely the dioxygenases, which are enzymes that have a strict catalytic requirement for both O_2_ and Fe^2+^ to carry out oxidoreductase activity (Aas et al. 2003; Falnes et al. 2004). As noted above, experimental data of AlkB activity on DNA and RNA demethylation reactions have been considered by Poole and Logan (2005) to raise the possibility that LUCA was in fact endowed with an RNA genome given the apparent RNA “repair” mediated by AlkB and other homologs.

Several arguments can be raised against this possibility. First, the experimental data have interpreted to argue that AlkB and its human homolog ALKBH3 are endowed with RNA “repair” activity. However, these experiments were performed with DNA and RNA homopolymers (Aas et al. 2003) that did not consider the complexity of the secondary structure of RNA present in mRNA and non-coding RNAs. Other assays have demonstrated lower efficiencies of AlkB and its homologs when the substrate is single-stranded or double-stranded RNA (van den Born et al. 2008). While the human homologue 3 (ALKBH3) and *E coli’s* AlkB have a strong preference for ssDNA, ALKBH3 (AlkB human homolog 3) also exhibits activity on viral single-stranded RNA genomes (Sedgwick et al. 2007; Falnes et al. 2004; Lee et al. 2005), but the recognition of the enzyme of the single-stranded nucleic acids is related to the regulation of mRNA and tRNA (Ougland et al. 2004, 2015). The differential preference and substate ambiguity may respond rather to the evolution of the homolog in a specific region of the cell like the nucleus (ALKBH2, ALKBH3, ALKBH4, ALKBH5), cytoplasm (ALKBH3) or even in mitochondria (ALKBH7) (Ougland et al. 2015).

In fact, the most likely explanation of AlkB and its homologs ambiguous recognition of substrates and the repair activity of RNA and DNA may lie in the chemical similarities of the single strand status of both RNA and DNA. RNA is typically a single-stranded molecule and DNA is momentarily single stranded during transcription and the replication process in phase S when it becomes highly susceptible to alkylation reactions (Falnes et al. 2002; Sedgwick 2004) and other chemical insults. Accordingly, the substrate ambiguity exhibited by AlkB and its homolog ALKBH3 may be a consequence of a similar substrate scaffold in single nucleic strands. The same phenomena probably explain the putative RNA replicase activity of a *Betaflexiviridae* virus (UNIPROT-A0A3G3LQ27_9VIRU) which includes a 2OG-FeII_Oxy_2 domain that recognises and binds the viral RNA during replications of these viruses.

The above arguments, combined with enzyme kinetics data of substrate preference in DNA instead RNA (Falnes et al. 2004), support our idea that the reported activity of AlkB (2OG-FeII_Oxy_2 domain) in methylated RNA is the outcome of an ambiguous substrate recognition. Our interpretation falls within the question raised by Sedgwick (2004) on the biological signification of RNA repair and support the possibility that RNA modifications may be related to complex RNA regulatory pathways of RNA molecules like mRNA and tRNA (Zheng et al. 2013; Roundtree et al. 2017; Boccaleto et al. 2022 and Wilkinson et al. 2022).

The evolutionary analyses of the structural superfamily cupin members (Figs. 2 and 3) demonstrates that the 2OGxs are a clearly defined monophyletic group (Fig. 3). The deeper node of these phylogenies suggest that the original cupin fold origin was not an oxidoreductase enzyme, but rather an anaerobic protein with transferase activity or with no enzymatic activity at all (see EC numbers in Fig. 3). A later node that indicates where the recruitment of O_2_ and Fe^2+^ may have evolved, represents an explosion of functions and divergence of the cupin fold. In particular, the oxidoreductase activity arises quickly in the neighbourhood of HgmA_N domain, but the diversification of enzymatic activity is not restricted only to oxidoreductases. Groups containing the JmjC domain and all the 2OGxs homologs reflect their late diversification of functions for eukaryotic histone (Klose et al. 2006) and demethylation of DNA.

The trees shown in Figs. 2 and 3 strongly suggests that the cupin fold domain may have emerged well before the free oxygen accumulation in the Earth atmosphere, and that the recruitment of molecular oxygen and iron necessary for an ancestral demethylation took place after the Great Oxidation Event ~ 2.4 Gyr once permanent concentrations of O_2_ was achieved (Lyons et al. 2014, 2024). In fact, the strict requirement of oxygen by dioxygenases allows insights on their emergence. Experimental data of the AlkB homolog 5 kinetics shows a 75% of reduced activity at 5.0% of atmosphere oxygen and a virtual absent activity at 1.0% of atmosphere oxygen (Thalhammer et al. 2011), so the requirement of free oxygen for its emergence and function may have had to be greater than 1% of atmospheric oxygen. Indeed, Jabłońska and Tawfik 2021 have suggested that enzymes that emerged after GOE they could have operated with in low oxygen levels.

The role performed by enzymes in pathways complexes provides an indirect way of relative dating of their emergence. For example, the role the 2OG-FeII_Oxy_3 and P4Ha_N domains in collagen posttranslational modifications in Metazoa is considered a milestone in the evolution and in fact, our results have shown that 2OG-FeII_Oxy_3 are in a very derivate node. Metazoan multicellularity requires the presence of collagen to maintain and create connective tissue. Collagen appears to be a strict requirement for the development of primordial interactions of cell aggregates in metazoa. Single-cell RNA sequencing has showed the expression of related enzymes of collagen posttranslational modifications in proline residues, like procollagen hydroxylase and collagen itself in larval and adult metacells of *Amphimedon queenslandica* (sponge), *Mnemiopsis leidyi* (ctenophore) and *Trichoplax adhaerens* (placozoa) (Sebé-Pedrós et al. 2018). By the same token, AlkB and its homologues functions should also be recent evolutionary development.

Proposals on the origin and early evolution of Metazoa rest on the idea that unicellular organisms with a complex life cycle evolved into multicellular organisms (Tikhonenkov et al. 2020; Sebé-Pedrós et al. 2018) but, until now, the origin of animals remains an open issue. A widely popular theory is the choanoblastaea theory, which assumes that the first animals were sponge-like entities, and that descended from a colony of choanoflagellate-like organisms, a possibility supported by the collar complex shared by choanoflagellates and sponges (Ruiz-Trillo et al. 2023). An alternative theory is the so-called synzoospore theory where the unicellular ancestor of animals was an organism with a complex life cycle and transitions between different life stages that fit with the current understanding of animal evolution (Ruiz-Trillo et al. 2023). Both of these explanations are based on the hypothesis of a common ancestor shared by choanoflagellates and the metazoa, but neither of them provides a detailed reconstruction of animal’s origins. As noted above, however, the metazoan ancestor was endowed with several key biological components required for multicellularity, including collagen biosynthetic encoding sequences.

As summarised by Sedgwick (2004), both AlkB and its homologue ABH3 can repair not only DNA but also RNA, albeit with a higher preference for the former. As she underlined, the same is true for other DNA repair mechanisms that have also been reported to act on RNA molecules, including *O*^*6*^*-*methylguanine-DNA-methyl transferase (Karran 1985) and the *E. coli* DNA-photolyase that repairs uracil cyclobutane dimers in poly(rU) molecules (Kim and Sancar 1991). These mechanisms simply reverse the damaged base and do not depend on the presence of a double-stranded nucleic acid to repair the excision of the modified nucleotide (Sedgwick 2004). Current estimates date the antiquity of LCA between 3.8 and 4.2 Gya ago (cf. Moody et al. 2024). As argued here, the available evidence strongly suggest that the AlkB-based repair mechanism could have not appeared during these early times, but are a much later evolutionary development once significant amount of free oxygen became available in the Precambrian environment. It is most likely that the use of RNA molecules as substrates for AlkB, the *O*^*6*^-methylguanine-DNA-methyl transferase (Karran 1985) and the *E. coli* DNA-photolyase (Kim and Sacar 1991) are the outcome of the lack of absolute specificity of these enzymatic mechanisms and cannot be used as indicating the presence of an RNA genome in the cenancestor.

## Supplementary Information

Below is the link to the electronic supplementary material.

Supplementary file1 (PNG 2481 KB) Fig. S1. Tertiary-based rooted phylogeny of all 72 domains/families endowed the cupin fold showing their biological distribution among the three cellular domains of life and some viruses. Tips marked with “E” indicates that this domain is present in the Eukarya, “B” in Bacteria, “A” in Archaea, and finally “V” indicates that the domain is present in some viruses
